# Are gait parameters altered in adults with hallux valgus?

**DOI:** 10.1186/1757-1146-6-S1-O29

**Published:** 2013-05-31

**Authors:** Sheree Nix, Bill Vicenzino, Natalie Collins, Michelle Smith

**Affiliations:** 1 School of Health and Rehabilitation Sciences, The University of Queensland, Brisbane, QLD, 4072, Australia; 2 School of Clinical Sciences, Queensland University of Technology, Brisbane, QLD, 4059, Australia; 3 Department of Mechanical Engineering, The University of Melbourne, Melbourne, VIC, 3010, Australia; 4 Department of Physiotherapy, The University of Melbourne, Melbourne, VIC, 3010, Australia

## Background

Gait parameters such as excessive pronation have been suggested as contributing to the development of hallux valgus (HV). HV has also been linked to functional disability in older adults. However, the literature investigating gait parameters in HV has not previously been systematically evaluated.

## Methods

A systematic review was conducted, searching electronic databases to October 2011. Cross-sectional studies with clearly defined HV and non-HV groups were included. Two investigators rated papers for methodological quality. Effect sizes (95% confidence intervals (CI)) were calculated as standardized mean differences (SMD) for continuous data and risk ratios (RR) for dichotomous data.

## Results

Nine papers were included (total n = 589). One study showed that during terminal stance HV participants had reduced ankle dorsiflexion (SMD = -0.81, CI: -1.44 to -0.18) and less rearfoot supination (SMD = -0.63, -1.25 to -0.01) compared to controls. In another study HV participants showed early onset of intrinsic muscle activity (RR 1.6, 1.1 to 2.2). Four studies investigating spatio-temporal parameters found no significant differences between groups, although one study found reduced speed (SMD -0.73), step length (SMD -0.66 to -0.59) and less stable gait patterns (SMD -0.86 to -0.78) in older adults with moderate to severe HV. Six studies investigated plantar pressures with inconsistent findings.

## Conclusion

Altered gait kinematics and muscle activity are apparent in HV, and these parameters warrant further investigation. Although conclusions regarding causality cannot be drawn from cross-sectional studies, interventions targeting these parameters may improve clinical outcomes in HV.

